# Editorial: Climate change and stress mitigation strategy in plants

**DOI:** 10.3389/fpls.2023.1291905

**Published:** 2023-09-28

**Authors:** Pawan Shukla, Anirudh Kumar, Rakesh Kumar

**Affiliations:** ^1^ Seri-Biotech Research Laboratory, Central Silk Board, Bangalore, India; ^2^ Department of Botany, Central Tribal University of Andhra Pradesh, Vizianagaram, India; ^3^ Department of Life Science, Central University of Karnataka, Kalaburagi, India

**Keywords:** abiotic stress, climate change, climate smart crop, genome editing, drought stress, high CO_2_

In the past five to six decades, anthropogenic activity has caused an unprecedented transformation in the global climate. It is anticipated that crops will be impacted by extreme climate change, such as a drought, heat stress, flood, or rise in sea level affecting crop production in coastal areas. These abiotic stressors alter the various molecular, biochemical, physiological and morphological parameters of plants, jeopardizing the global food and nutritional security, especially in underdeveloped and developing nations (Shukla et al., 2022).

In response to abiotic stress, plants have evolved specialised coping mechanisms that enable them to thrive in various environments (Sharma et al., 2022) (**Figure 1**). These tactics result from plants evolutionary adaptations to flourish under varied environmental conditions. Multiple genes linked to various stress-related pathways are eventually expressed in response to any environmental change signal in order to combat abiotic stress (Ahmed et al., 2021).

**Figure 1:**
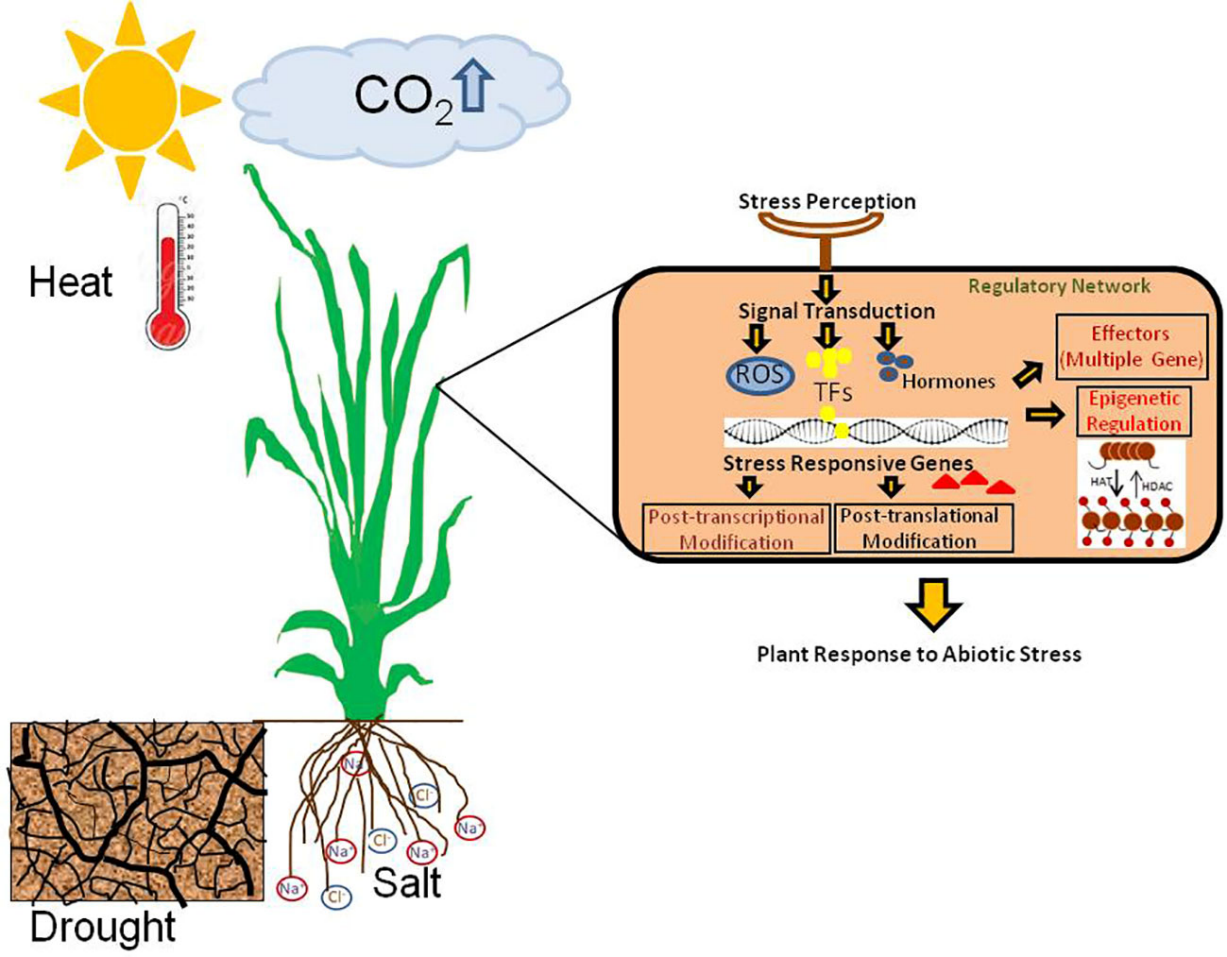
Abiotic stress response mechanisms in plants.

Frequent environmental changes have a major influence on the vegetative development of plants from a range of perspectives, leading to molecular, cellular, physiological, and morphological changes (Patel et al., 2021). Therefore, it is important to decipher the abiotic stress response mechanisms in plants in order to develop tailor-made crops that can cope with climate change. The advent of Omics tools has revolutionized the agriculture field. The genomics, transcriptomics, proteomics and metabolomics studies conducted under various abiotic stress conditions on numerous agriculturally significant crop plants have increased our understanding of the mechanisms underlying stress tolerance (Gupta et al., 2021; Singh et al., 2021).

This research endeavor, encapsulated within the Research Topic, aims to curate the most recent advancements and provide insights into the evolving landscape of research focused on unraveling the fundamental intricacies of stress signaling and adaptation mechanisms inherent to plants. It endeavors to comprehensively explore the diverse spectrum of abiotic stresses and their influence, while also delving into their application within the realm of crop management and enhancement, aligning with the overarching goal of fostering the growth of climate-resilient crops.

The selected articles in this Research Topic collectively offer a comprehensive panorama of diverse research perspectives on plant responses to various abiotic stresses. Research involving Abscisic acid-Polyacrylamide (ABA-PAM) treatment has shown promise in enhancing the rhizosphere microbial community and bolstering forage plants’ resilience against drought. This significant study offers potential solutions for addressing drought-induced grassland degradation. Additionally, salinity stress represents a critical abiotic challenge hindering plant growth. An enlightening review article delving into plant adaptation strategies to combat salinity stress via osmolyte accumulation, coupled with its interaction with phytohormones, not only enhances our comprehension but also guides the development of climate-resilient crops.

Histone deacetylase play an important role in regulation of gene expression through deacetylation. The investigation on histone deacetylase 2 (HD2) protein family in cotton genome revealed their evolutionary relationship and higher expression of GhHDT3D.2 gene in response to drought and salt stress. This study not only sheds light on the evolutionary trajectory of cotton’s HD2 genes but also establishes a foundation for future investigations into enhancing drought and salinity stress resilience in cotton.

DnaJ type-I protein belongs to the heat shock protein family, recognized for its pivotal role in protein folding, protein repair and their reactivation—a cornerstone of stress tolerance mechanism in plants. The heterologous expression of DnaJ type-I protein- a homolog of *Vigna aconitifolia* (*Va*DJI), revealed its role in ABA insensitivity and multiple stress tolerance in transgenic tobacco plants. This revelation sheds light on VaDJI’s mechanisms, elucidating its positive control over drought stress tolerance and ABA signaling—a valuable asset for enhancing drought and heat stress tolerance in crops. Further, the advent of gene editing tool has revolutionized the area of genetic manipulation in plants. A comprehensive review article summarizes the recent breakthroughs, unraveling abiotic stress response mechanisms in plants and unveiling the potency of the CRISPR/Cas-mediated gene-editing system. A spectrum of stressors-drought, salinity, cold, heat, and heavy metals—finds enhanced tolerance through this revolutionary approach. Notably, the review underscores the cutting-edge strides in prime editing and base editing tools, underscoring their potential in crop improvement.

A comprehensive exploration of groundnut’s response to heat stress has yielded a noteworthy article. This study identifies genomic regions and candidate genes associated with heat tolerance related traits. Impressively, the investigation uncovered 45 major main effect QTLs controlling 21 agronomic traits. Notably, the research unraveled the presence of three QTL clusters (Cluster-1-Ah03, Cluster-2-Ah12, and Cluster-3-Ah20), harbouring a substantial proportion of the major QTLs (30 out of 45, or 66.6%). These clusters accounting 10.4% to 49.5% phenotypic variance. Further, this study spotlighted important candidate genes within these QTL clusters. These genes include the *DHHC-type zinc finger family protein* (*arahy.J0Y6Y5*), FRIGIDA-like protein (arahy.0C3V8Z), *Kelch repeat F-box protein* (*arahy.I7X4PC*), *Pentatricopeptide repeat-containing protein* (*arahy.4A4JE9*), *peptide transporter 1* (*arahy.8ZMT0C*), and *post-illumination chlorophyll fluorescence increase* (*arahy.92ZGJC*) and *Ulp1 protease family* (*arahy.X568GS*). Their involvement underscores the genetic basis of heat stress adaptation in groundnut. This study provides a platform for further fine mapping, gene discovery, and the development of markers for genomics-assisted breeding aimed at developing heat-tolerant superior lines/varieties.

Moreover, a compelling synergy between elevated CO_2_ (eCO_2_) and selenium nanoparticles (SeNPs) was unveiled in wheat plants. Notably, the combined effects of selenium nanoparticles and eCO_2_ surpassed the effect of eCO_2_ alone. The treatment of SeNPs + eCO_2_ yielded remarkable benefits, evidencing enhanced total antioxidant capacity, polyphenols, flavonoids, and total tocopherols within plants. This treatment also culminated in the highest accumulation of photosynthetic pigment content in leaves. While eCO_2_ alone exhibited positive effects on Rubisco activity and stomatal conductance, this study fundamentally illuminates the intricate interplay between climate change impacts and crop responses, highlighting the potential of nanoparticle treatments.

Overexpression of peanut (*Arachis hypogaea* L.) *AhGRFi* gene in *Arabidopsis thaliana* led to an enhanced suppression of root growth under exogenous NAA treatment. In addition, this overexpression triggered an upregulation of auxin-responsive genes including *IAA3, IAA7, IAA17*, and *SAUR-AC1*, while genes *GH3.2* and *GH3.3* experienced downregulation in transgenic plants. Notably, the response of genes *GH3.2, GH3.3*, and *SAUR-AC1* showcased contrasting shifts under NAA treatment. These findings collectively indicate the involvement of the 14-3-3 protein in peanut in modulating root growth through intricate auxin signaling pathways.

## Conclusions and perspective

The abiotic stress response mechanism in plant is a complex process. This amalgamation of studies significantly advances our understanding of plant responses to abiotic stresses. This Research Topic offers promising avenues for enhancing crop resilience and productivity amidst the dynamic shifts in our climate/in the face of changing climates. We believe that this Research Topic encourages future research in this challenging but vital and fruitful field of study.

## Author contributions

PS: Writing – original draft, Writing – review & editing. AK: Writing – review & editing. RK: Writing – review & editing.
